# The Effects of a Varied Gold Shell Thickness on Iron Oxide Nanoparticle Cores in Magnetic Manipulation, T_1_ and T_2_ MRI Contrasting, and Magnetic Hyperthermia

**DOI:** 10.3390/nano10122424

**Published:** 2020-12-04

**Authors:** Grace Brennan, Silvia Bergamino, Martina Pescio, Syed A. M. Tofail, Christophe Silien

**Affiliations:** Department of Physics and Bernal Institute, University of Limerick, Limerick V94 T9PX, Ireland; grace.brennan@ul.ie (G.B.); silviabergamino@gmail.com (S.B.); martina.pescio@gmail.com (M.P.); tofail.syed@ul.ie (S.A.M.T.)

**Keywords:** magnetic-plasmonic nanoparticles, nuclear magnetic resonance, magnetic hyperthermia, magnetic drug delivery, gold shell, magnetic manipulation, nanotheranostics

## Abstract

Fe_3_O_4_–Au core–shell magnetic-plasmonic nanoparticles are expected to combine both magnetic and light responsivity into a single nanosystem, facilitating combined optical and magnetic-based nanotheranostic (therapeutic and diagnostic) applications, for example, photothermal therapy in conjunction with magnetic resonance imaging (MRI) imaging. To date, the effects of a plasmonic gold shell on an iron oxide nanoparticle core in magnetic-based applications remains largely unexplored. For this study, we quantified the efficacy of magnetic iron oxide cores with various gold shell thicknesses in a number of popular magnetic-based nanotheranostic applications; these included magnetic sorting and targeting (quantifying magnetic manipulability and magnetophoresis), MRI contrasting (quantifying benchtop nuclear magnetic resonance (NMR)-based T_1_ and T_2_ relaxivity), and magnetic hyperthermia therapy (quantifying alternating magnetic-field heating). We observed a general decrease in magnetic response and efficacy with an increase of the gold shell thickness, and herein we discuss possible reasons for this reduction. The magnetophoresis speed of iron oxide nanoparticles coated with the thickest gold shell tested here (ca. 42 nm) was only ca. 1% of the non-coated bare magnetic nanoparticle, demonstrating reduced magnetic manipulability. The T_1_ relaxivity, r_1_, of the thick gold-shelled magnetic particle was ca. 22% of the purely magnetic counterpart, whereas the T_2_ relaxivity, r_2_, was 42%, indicating a reduced MRI contrasting. Lastly, the magnetic hyperthermia heating efficiency (intrinsic loss power parameter) was reduced to ca. 14% for the thickest gold shell. For all applications, the efficiency decayed exponentially with increased gold shell thickness; therefore, if the primary application of the nanostructure is magnetic-based, this work suggests that it is preferable to use a thinner gold shell or higher levels of stimuli to compensate for losses associated with the addition of the gold shell. Moreover, as thinner gold shells have better magnetic properties, have previously demonstrated superior optical properties, and are more economical than thick gold shells, it can be said that “less is more”.

## 1. Introduction

Inorganic nanomaterials such as gadolinium [1], iron oxide [2], silica [3], and gold [4] are becoming increasingly popular in nanotheranostic applications (combined therapeutics and diagnostics), particularly as imaging agents, drug deliverers, and hyperthermia agents. It is hypothesized that combining materials into a single nanoplatform strengthens the physical properties of the nanosystem. One such example is the combination of a magnetic and plasmonic material into a single hybrid nanostructure. Ideally, this magnetic-plasmonic nanoparticle will maintain the physical properties of each material when combined in the bi-functional nanomaterial. A plasmonic gold shell facilitates a number of optical-based applications, including surface-enhanced Raman scattering (SERS) [5], photothermal activity [6], and optical imaging contrast [7]; and these applications could be combined with magnetic-based applications if a magnetic core is used. For this study, we measured how a gold shell grown on a magnetite core affects the nanoparticle efficacy in various magnetic-based nanotherapeutic applications to understand the implications of magnetic-plasmonic nanoparticle hybridization. We found that, as prepared, the core–shell nanoparticle remains meaningfully efficient in magnetic-based applications for gold thicknesses of approximately ≤4 nm with iron oxide cores of ca. 20 nm.

Magnetic nanomaterials are at the forefront of inorganic nanotheranostics, particularly in diagnostics as contrast agents in nuclear magnetic resonance (NMR)-based applications, including magnetic resonance imaging (MRI) [8] and NMR-based biosensing [9], and in therapeutics as drug delivery [10,11] and hyperthermia agents [12]. The ability to manipulate or interact with material at the nanoscale with external stimuli is of utmost interest [13]. Magnetic nanomaterials may be manipulated by an external magnetic field, facilitating magnetic flux-driven drug delivery [10] for therapeutics, and magnetic separation of cells or blood [14] for diagnostics. Many types of magnetic nanoparticles exist, but superparamagnetic iron oxide (magnetite) nanoparticles are arguably the most prevalent in nanotheranostics [15]. Superparamagnetism is a type of magnetism that exists in nanoscale single magnetic domain ferri- or ferromagnetic materials. Superparamagnetic nanoparticles have two stable antiparallel orientations of the magnetic moment, with an energy barrier separating these orientations. In the absence of an external magnetic field and at temperatures where the energy barrier is less than the thermal fluctuation energy, the magnetic moment regularly flips direction leading to a net magnetization averaged to zero. In other words, superparamagnetic nanoparticles exhibit a net zero magnetic moment at certain temperatures in the absence of a magnetic field. However, when a magnetic field is applied, the magnetic moments align with the field, acting like a paramagnet but with higher magnetic susceptibility. Non-superparamagnetic magnetic nanoparticles experience strong interparticle magnetic forces in the absence of an external field that leads to aggregation. By contrast, superparamagnetic nanoparticles do not experience these strong interparticle magnetic forces in the absence of a magnetic field, and thus may be colloidally stable, a big advantage in many applications.

Along with superparamagnetic iron oxide nanoparticles, plasmonic nanoparticles are popular in the field of nanotheranostics [16,17,18]. A plasmon is a collective oscillation of the local electron density of a material; near optical resonance, this local electron density coherently oscillates with incident electromagnetic (light) excitation. This leads to enhanced optical absorption, scattering, and local electric field, which can be exploited in several applications including imaging [19,20] and sensing [21] and in drug delivery [22]. Furthermore, similar to magnetic nanomaterials, they are effective hyperthermia agents in plasmonic photothermal therapy [23]. When these plasmonic nanoparticles are excited by light near or at their optical resonance frequency, the mobile carriers dissipate their energy in the form of heat, which is then diffused into the surrounding area. Gold is typically chosen as the plasmonic material in bioapplications due to its stability, plasmon resonance located in the visible/near-infrared (NIR), and biocompatibility [24].

Many geometries of magnetic-plasmonic nanostructure exist, but to take advantage of the superior stability and biocompatibility of gold, the exterior should be gold, i.e., a core–shell structure. Magnetite–gold core–shell nanoparticles have also been used for photothermal activity [25], SERS enhancement [26], optical imaging contrast [27], interesting optical effects [28], enhanced Faraday rotation [29], and optical modulation in low-field magnetic relaxation [30] and have demonstrated good biocompatibility [31]. The gold shell in these reports varies dramatically, from thin or incomplete to thick gold shells. Ideally, such magnetic-plasmonic nanoparticles could be used in combined nanotheranostic applications, for example, photothermal therapy in combination with MRI imaging, simultaneous optical imaging and magnetic hyperthermia, or magnetic separation combined with SERS. From our previous work [28], we found that thicker gold shells led to increased light scattering, and we would also expect decreased cytotoxicity [32] and circulation half-life [33] for this nanostructure. Meanwhile, thin-to-moderate gold shells facilitated spectrally selective photothermal activity due to the emergence of a spectral drift [28]. For this study, we explored the effects of the plasmonic gold shell in three notable magnetic-based applications, by comparing five different stages of magnetic-plasmonic nanoparticle with varied gold shell thickness. The three popular nanotheranostic applications considered were magnetic separation and targeting ability (by quantifying magnetophoresis), T_1_ and T_2_ MRI contrasting (by quantifying NMR relaxivity), and magnetic hyperthermia activity (by monitoring the heat generation in an alternating magnetic field).

## 2. Materials and Methods

### 2.1. Synthesis of Multistage Magnetic-Plasmonic Nanoparticles

First, 20.5 ± 1.3 nm diameter commercial oleic acid-capped iron oxide nanoparticles (Ocean NanoTech, San Diego, CA, USA) were amine functionalized by ligand exchange using (3-aminopropyl) triethoxysilane (APTES) and using a protocol modified from [34], to aid further functionalization and improved biocompatibility [35] (denoted O, black in graphs). Next, tetrakis(hydroxymethyl) phosphonium chloride (THPC)-reduced gold nanoseeds prepared by the Duff method [36] were grafted onto the iron oxide core giving a core–satellite or seeded stage (Os, red). The gold-seeded iron oxide nanoparticles were then subjected to three iterative gold reductions using a gold-plating solution and formaldehyde reduction modified from [37], yielding three further stages of gold growth called R1 (green), R2 (blue), and R3 (orange) throughout, and had a diameter determined by TEM of 28.5 ± 2.2, 42.1 ± 10.3, and 103.7 ± 16.9 nm, respectively. To read further details about the synthesis of these multistage magnetic-plasmonic nanostructures and see transmission electron micrographs of the various stages, please see our earlier work in [24]. To aid the comparison between gold-coated stages and uncoated iron oxide, the mass of iron oxide (i.e., the number of nanoparticles) was kept constant in this work.

### 2.2. Magnetophoresis

To conduct optical tracking of magnetophoresis, a deuterium–halogen UV–VIS–NIR light source (DH2000-BAL, Ocean Optics, Orlando, FL, USA) using only halogen as the light source was passed through a quartz cuvette (CV10Q3500, Thorlabs, Newton, NJ, USA) containing the suspended nanoparticles. The transmitted light was focused onto a flame spectrometer (Ocean Optics), which was paired with OceanView software (Version 1.6.7, Ocean Optics). The probed area was a cylindrical region in the cuvette with a 5 mm diameter and a 10 mm height, centered 70 mm above a magnet (neodymium, 10 mm × 10 mm × 10 mm). The magnet’s distance-dependent magnetic flux density was measured using a gaussmeter. The extinction peak was monitored temporally, and a linear fit of the peak extinction is used to monitor magnetophoresis.

### 2.3. NMR T_1_ and T_2_ Relaxation Measurements

To conduct T_1_ and T_2_ measurements of the multistage magnetic-plasmonic nanostructures, a benchtop NMR (^1^H Spinsolve 60 MHz, Magritek, Malvern, PA, USA) was used. The magnetic field strength was 1.5 Tesla, and the proton Larmor frequency was 60MHz. To track the effect of the gold shell on the magnetic nanoparticle relaxivity, the ^1^H (water) peak at ≈4.8 ppm was monitored.

T_1_ measurements were conducted using an inversion recovery pulse sequence, starting with the shortest inversion delay; a series of experiments were conducted with the inversion delay stepped linearly to the maximum inversion time. Two scans of 1.6 s acquisition time (8192 points, dwell time of 200 μs), repetition time of 7 s, and a varied inversion time of ≈5 s with 21 steps were taken for the T_1_ measurements.

T_2_ measurements were made using a Carr–Purcell–Meiboom–Gill (CPMG) pulse sequence, starting with the shortest echo time τ; a series of measurements were conducted where 180-degree pulses were stepped linearly, ending with the final (variable) echo time. Four scans with an acquisition time of 0.4 s (8192 points, 50 μs dwell time), 4 s repetition time, 20 steps with a CPMG echo time of 0.2 ms, and a varied final echo time of ≈2 s were taken for the T_2_ measurements. The 90° pulse width was 7 ms for both measurements.

### 2.4. Magnetic Hyperthermia

In this research, a Flexitune induction heater (Radyne, Wokingham, UK) was used with a water-cooled homemade copper coil, generating a 100 mT alternating magnetic field, with a frequency of 20 kHz. Induction heaters are typically used to heat a conductive material by eddy currents. Induction heaters are simply AC passed through an inductor (e.g., coil), thus producing an alternating magnetic field, per Ampere’s law. A calibrated A655sc (FLIR, Wilsonville, OR, USA) infrared camera was used to monitor the magnetic field-induced hyperthermal activity, noncontact. Aqueous solutions of the various stages of magnetic-plasmonic nanoparticles in an Eppendorf tube (Eppendorf, Hamburg, Germany) (at a fixed concentration of 0.2 mg/mL of iron oxide) were suspended inside the cooled copper coil (noncontact) using a plastic mount. It is important to note that these measurements were not conducted under adiabatic conditions (i.e., there was a loss to the environment) unlike many other magnetic hyperthermia measurements [38], which are thermally insulated.

## 3. Results

**Figure 1:**
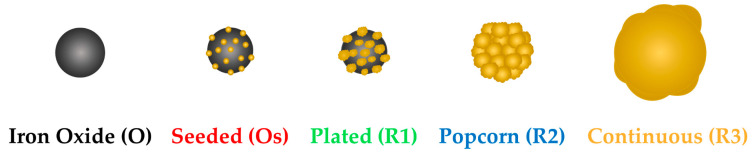
Illustration of the various forms and thicknesses of magnetic-plasmonic nanoparticles studied for this paper. From left, the (3-aminopropyl) triethoxysilane (APTES) functionalized iron oxide (Fe_3_O_4_), called O throughout. Next, the Fe_3_O_4_ nanoparticles functionalized with gold seeds, Os. Middle, the Fe_3_O_4_–Au gold-plated nanoparticle after one gold reduction, R1. R2 is the magnetic-plasmonic nanoparticle after two gold reductions and, lastly, R3, after three gold reductions. Reproduced with permission from [28]; Copyright The Royal Society of Chemistry, 2020.

To determine whether a gold coating affects the efficacy of magnetic nanoparticles in various magnetic-based nanotheranostic applications, Fe_3_O_4_–Au nanoparticles were synthesized with various stages of gold growth (see Section 2.1 for details). An illustration of the various nanoparticle stages is shown in Figure 1.

### 3.1. Magnetophoresis

Magnetic guidance or translation of magnetic (or hybrid magnetic) nanoparticles is vital in facilitating magnetic flux-driven drug delivery, magnetic cell or blood sorting, and magnetic field-guided therapy. Magnetic nanoparticles suspended in fluid travel toward the highest magnetic field gradient; this movement is called magnetophoresis. A higher magnetophoresis speed is preferable for most applications and has been achieved by using a stronger magnetic field gradient, higher nanoparticle magnetization or concentration, nanoparticle shape anisotropy [39], and surface charge [40]. Considerable work in the magnetophoresis of various magnetic (and magnetic-plasmonic) structures has been carried out [41,42,43]. Furthermore, the addition of a nonmagnetic silica [44,45] and polymer [43] shell has been shown to decrease the magnetophoretic mobility of magnetite nanoparticles. Thus, if a polymer/silica shell affects the magnetophoresis, it is reasonable to hypothesize that a gold shell would also hamper this motion.

The magnetophoretic force, *F_M_*, exerted on a magnetic particle by an external magnetic field is dependent on the magnetic particle volume, *V_p_*, the magnetization of the nanoparticle, M_p_, the permeability of free space, μ_0_, and the magnetic field strength, H, at the particle location per [46,47]:

*F_M_* = *μ_0_V_p_*M_p_. ∇H (1)

Equation (1) is sufficient for non-interacting (noncooperative) high magnetic field gradient magnetophoresis. Drag and magnetic interaction forces should be considered in low magnetic field gradient magnetophoresis. For low magnetic field gradient (<100 T/m) magnetophoresis, as is the case here, typically magnetic particles cooperate by clustering together into chain-like structures while moving in the gradient, and this assembly accelerates the magnetophoresis transport [48,49]. This is likely due to the coupling of the individual magnetic moments of the nanoparticles into a new and larger effective magnetic moment. Furthermore, the drag force (friction) experienced by the assembled nanoparticles would be reduced per the slender-body theory [50]. Magnetic dipolar interaction forces that facilitate this magnetic assembly are increased when the nanoparticles are not colloidally stable and are in high concentrations (due to increased probability of proximity).

The magnetic saturation of Fe_3_O_4_–Au nanoparticles has been reported to be decreased compared to uncoated Fe_3_O_4_ by varying degrees from ≈10% to 70% [51,52,53,54,55,56], likely a result of increased gold-to-iron oxide mass ratio and magnetic shielding by the diamagnetic gold shell [57,58,59]. This reduced magnetic saturation has also been reported for other materials with increased shell thickness, including gold shells on cobalt nanoparticles [60] and silica shells on FeNi nanoparticles [61]. On the contrary, there are reports of increased magnetic saturation with the addition of a gold shell on Fe_3_O_4_ [62,63], and the authors hypothesized that conduction electrons are trapped, which induces an orbital moment. A reduction of magnetic saturation reduces the value M_p_ can reach, thus leading to a reduction in the magnetic force causing the translation of the nanoparticles to the magnet. As the gold shell on the Fe_3_O_4_ core was associated with a reduced saturation, we anticipated a reduced magnetic force and magnetophoresis.

**Figure 2:**
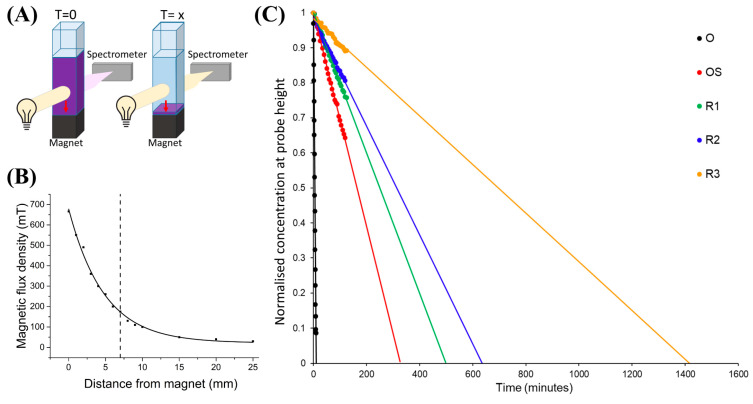
(**A**) The experimental setup to monitor the magnetophoretic behavior, using a white light source and spectrometer to monitor the diffusion of the nanoparticles to a permanent magnet. (**B**) The distance-dependent magnetic flux density of the permanent magnet used in this experiment, with the dashed line representing the probe height. (**C**) The magnetophoresis of the various stages of the magnetic-plasmonic nanoparticles. On the vertical axis, 1 represents the starting concentration of 20 μg/mL of Fe_3_O_4_. The data are extrapolated to estimate the time for total magnetophoresis out of the probed area.

In this work, the magnetophoresis of the multistage magnetic-plasmonic nanoparticles was monitored by optical extinction, per the experimental schematic seen in Figure 2A. In this experiment, the concentration of iron oxide (and thus, the number of nanoparticles) was kept constant. Therefore, the volume of magnetic material is constant, but the percentage per particle varies with each stage (see Table 1). Collimated light is passed through the cuvette containing the nanoparticles, which is then focused onto a fiber spectrometer. A permanent magnet is placed below the cuvette, with a distance-dependent magnetic flux density seen in Figure 2B. The probed area is a cylindrical region in the cuvette with a 5 mm diameter and a 10 mm height, centered 70 mm above the magnet. As the nanoparticles migrate toward the magnet (due to magnetic forces) and leave the probe area, the optical extinction decreases. The peak optical extinction was tracked during magnetophoresis and a linear fit was added (see Figure 2C). Further experimental details are given in the experimental Section 2.1.

From Figure 2C and Table 1, increasing the gold shell thickness leads to a significantly longer time to magnetically separate, i.e., decreased speed of magnetophoresis. The iron oxide nanoparticles without any gold coating take approximately 12 min to migrate out of the probed area of the solution. With the addition of gold, the magnetic separation time increases, eventually reaching 1421 min for the thickest gold shell. Thus, thinner gold shells are likely preferable for applications that require reasonable responsivity to the external magnetic field. Alternatively, much larger magnetic field gradients would be required to use these thick gold-shelled nanoparticles in such applications. It is likely a combination of a reduced magnetic saturation and loss of cooperative behavior of the thickly gold-coated nanoparticles that leads to reduced magnetophoresis. Furthermore, an increased drag force would be experienced by larger nanoparticles.

It should be noted that the surface charge of the nanoparticles may also affect the interaction between nanoparticles. Zeta potential measurements of the various stages of nanoparticle are given in Table 1, indicating good colloidal stability for R1, R2, and R3. Temperature and pH were constant throughout.

### 3.2. NMR/MRI Contrast Agent

MRI is a well-known non-ionizing technique that uses radio waves along with strong magnetic fields and gradients to conduct biological diagnostic imaging. MRI imaging is based on NMR principles, where certain atomic nuclei (typically hydrogen) undergo Larmor precession in a strong external magnetic field and, when the Larmor precession frequency of the nuclear magnet resonates with that of an incoming radio wave (RF pulse), energy is absorbed from the radio wave. Protons may align in two energy eigenstates, low and high energy, separated by a small splitting energy. At equilibrium, most protons reside in the low energy state (aligned with the external magnetic field) and a net polarization parallel to the external field. An incident resonant RF pulse may lead to the polarization vector either tipping sideways (90° pulse) or reversing (180° pulse), and the protons come into phase with the pulse and one another (phase coherence). In response to the RF pulse, we may consider two relaxations to equilibrium, namely spin–lattice (also known as T_1_ or longitudinal relaxation) and spin–spin (also known as T_2_ or transverse relaxation). The recovery of the longitudinal magnetization relaxation (M_z_) to its thermal equilibrium value (M_z,eq_) is called the T_1_ (spin–lattice) relaxation; as the T_1_ relaxation is related to the redistribution of the spins to the low energy state (thermal equilibrium), energy is dissipated to the surroundings (lattice). T_2_ relaxation involves the recovery of the equilibrium state dephased spin; therefore, the magnetization vector perpendicular (transverse) to the static magnetic field is considered, M_xy_.

A downfall of MRI biological imaging is its low sensitivity. To combat low sensitivity, a contrast agent is commonly employed to selectively alter the relaxation time of the nuclei. As the magnetic relaxation of the nuclei varies with the square of the magnetic dipole moment, gadolinium (Gd^3+^) with its seven unpaired electrons (high magnetic moment) is often used in chelated form to enhance MRI T_1_ contrast. However, there are concerns regarding the safety of these gadolinium-based contrast agents [64]. Superparamagnetic nanoparticles have been proposed as an alternative, but rather mostly for T_2_ image contrast.

In terms of MRI, a local reduction (shorter time) in either the T_1_ relaxation (positive contrast) or T_2_ relaxation (negative contrast) results in better image contrast. As the T_1_ and T_2_ times are concentration-dependent, we define relaxivities as a function of iron concentration, i.e., r_1_ and r_2_. The longitudinal relaxivity r_1_ is said to depend on the molecular tumbling time, proton residence lifetime, and coordinating number, while the transverse relaxivity r_2_ is proportional to the square of the magnetic nanoparticle radius and the magnetic saturation [65]. As the T_1_ relaxation is associated with inner-sphere mechanisms (chemical energy exchange), superparamagnetic nanoparticles are instead more effective in T_2_ applications.

In the case of magnetic core–nonmagnetic shell particles, the magnetic field experienced by the water protons (T_2_) and the degree of chemical exchange (T_1_) lessens with increased shell thickness. Furthermore, if the nanoparticles are aggregated or assembled, worse r_1_ contrast due to reduced surface area and improved r_2_ contrast owing to the coupling of the magnetic moments are expected [66]. A relaxivity ratio (r_2_/r_1_) is often used as another measure of contrast agent usefulness. An optimal negative contrast agent (T_2_) would have a high r_2_/r_1_ ratio along with a high r_2_ value. If this ratio is less than 5, the contrast would instead be considered a good candidate for a T_1_ contrasting; if larger than 5, the agent may be more suitable for T_2_ contrasting [67].

Several r_1_ and r_2_ relaxivities from the literature are presented in Table 2, as well as the values for the multistage magnetic-plasmonic structures from this work. Marangoni et al. [68] studied the T_1_ relaxation of gold-core nanoparticles with a Gd(III) ion–silica layer, followed by a varied gold shell. Interestingly, T_1_ contrast was better for the gold-based nanoparticles compared to the typical Gd-based chelating agents. They also found that contrast was optimal for a gold-seeded shell but was seen to decrease with additional gold growth. The authors suggest that the added mass from the shell decreases the tumbling rate, hence decreasing the relaxivity for the thicker shelled nanoparticles (worse contrast). Pinho et al. [69] studied the effect of silica shell thickness on the r_1_ and r_2_ relaxivities of γ-Fe_2_O_3_ (see Table 2 for details). The thickness of silica had a significant impact on both relaxivities, with increased shell thickness leading to a decrease in the relaxivity value (decreased contrast). Others show the decrease in r_1_ [70] or decrease in both r_1_ and r_2_ [69,71] with the addition of a SiO_2_ shell, similar to observations in this research with the addition of a gold shell. Moreover, Park et al. noted a reduced r_2_ value for a diamagnetic avidin–biotin coating on Fe_3_O_4_ nanoparticles and attributed this to diamagnetic shielding effects [72]. Assembled magnetic structures were also seen to increase the r_2_ relaxation [73]. In this work, benchtop ^1^H NMR spectroscopy was used to monitor the effects of the multistage magnetic-plasmonic nanoparticles on the relaxation of the water associated peak at ca. 4.8 ppm. Information on the T_1_ and T_2_ measurement sequences can be found in the experimental Section 2.3.

**Figure 3:**
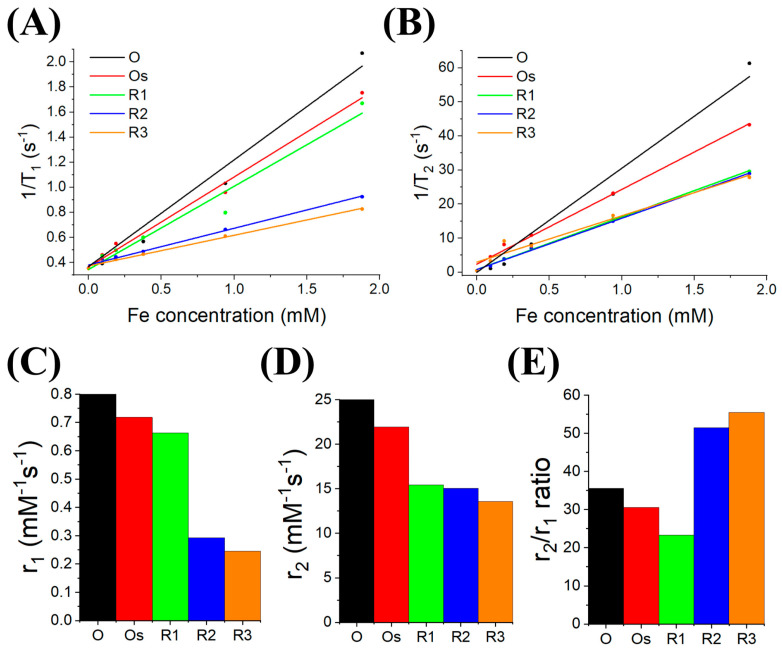
Nuclear magnetic resonance (NMR) of the various stages of magnetic-plasmonic nanostructures. (**A**) shows the T_1_ relaxation time (s^−1^) of the various nanostructures at different concentrations of iron (mM). (**B**) shows the graph of the T_2_ relaxation time with varied concentrations of the multistage nanoparticles. (**C**,**D**) are bar graphs of the relaxivities r_1_ and r_2_, respectively. (**E**) shows the r_2_/r_1_ ratio of the magnetic-plasmonic nanostructures.

From the data presented in Figure 3 and Table 2, we can conclude that the added gold has a considerable impact on the relaxation properties of the iron oxide nanoparticles. Beginning with T_1_ properties, the iron oxide nanoparticles (O) have a r_1_ relaxivity of 0.92 mM^−1^ s^−1^; this reduces with added gold to 0.24 mM^−1^ s^−1^ for the thick gold-shelled iron oxide nanoparticle (R3). In the case of with T_2_ relaxation, there is also a reduction in the r_2_ relaxivity, which is 32.70 mM^−1^ s^−1^ for the bare iron oxide (O) and 13.58 mM^−1^ s^−1^ for the iron oxide core with the thick gold shell (R3). Interestingly, the r_2_/r_1_ ratio is minimum for the iron oxide nanoparticle with a thin gold shell (23.27) and maximum for the thick gold-shelled iron oxide nanoparticle (55.48). This large ratio for the thick gold shell could suggest that this stage is a good candidate for T_2_ imaging, but it is not paired with a high r_2_ value. In summary, increasing the thickness of the gold shell reduces the relaxivity (increases the relaxation time) for both T_1_ and T_2_ MRI imaging, hence reducing the contrasting capability.

### 3.3. Magnetic Hyperthermia

Hyperthermia therapy is a medical treatment that involves raising the temperature of the body in an attempt to treat diseases such as cancer. Standard hyperthermia therapy may be delivered using infrared light, microwaves, or radiofrequency. Like with other cancer treatments, there are adverse effects when non-targeted tissue is affected, which can lead to blood clots or even cardiovascular issues [77]. The temperature to achieve cell death is debated, although most agree that 40–43 °C must be reached, about 3–6 °C above healthy body temperature (37 °C) [77]. It is important to note that such therapy is not a conventional therapy, and would rarely be used as the first choice for treatment due to non-selective heating. To combat issues with non-selective heating, nanoparticle agents have been proposed as a technique for localizing the heat to targeted areas with minimal heating of the surrounding healthy tissue. One such nanoparticle agent is magnetic nanoparticles. Magnetic hyperthermia combines magnetic nanoparticles with an external high-frequency alternating magnetic field. To achieve selective heating, the nanoparticle should target the tumor site, using either passive or, preferably, active targeting techniques (using targeting ligands, magnetic field).

Typically, superparamagnetic nanoparticles are used as magnetic hyperthermia agents. Superparamagnetic nanoparticles flip to align their magnetic dipole to the external magnetic field orientation. When this magnetic field is alternating, and at a high frequency, these nanoparticles flip to orient with the field but lose power due to hysteresis, resulting in heat dissipation.

In an AC magnetic field, the magnetization of the nanoparticle may not be responsive enough to keep up with the AC frequency, resulting in a phase difference. This phase difference requires a complex magnetic susceptibility explanation. The concentration, size, and material of the nanomaterial impact the hyperthermia activity along with the field strength and frequency. Furthermore, magnetic assembly leads to higher hysteresis losses, thus increasing the heat generated [78]. A silica shell [79,80] and a polyphosphazene–gold shell [81] have also been shown to decrease magnetic hyperthermia activity.

To benchmark the heating efficiency of the magnetic-plasmonic nanoparticles, the specific power absorption (SPA) (W/g), also known as the specific absorption rate (SAR), is calculated per [82]. Furthermore, to account for the magnetic field strength and frequency used, the intrinsic loss power parameter (ILP) (H m^2^ kg^−1^) is calculated per [82].

**Figure 4:**
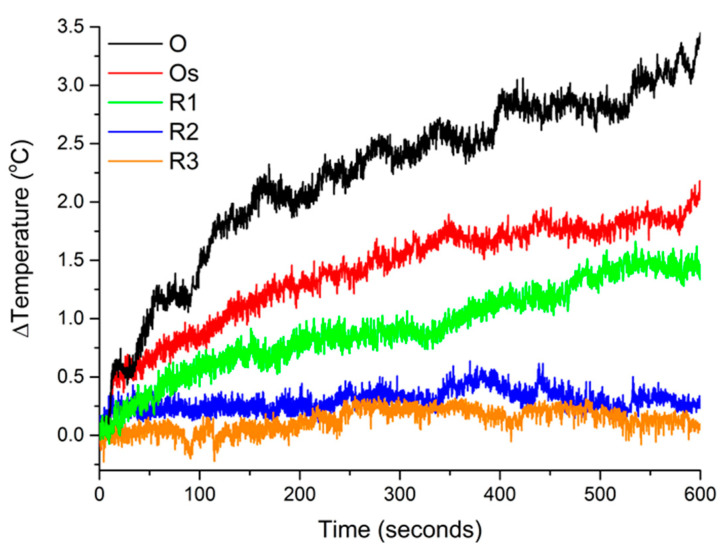
Magnetic hyperthermia activity of the various magnetic-plasmonic nanostructures all at a concentration of 0.2 mg/mL of Fe_3_O_4_.

From Figure 4 and Table 1, we can deduce that the addition of gold leads to a reduction in the magnetic hyperthermia activity. As previously discussed, it is likely that with the addition of gold there is a reduction of interparticle assembly as this formation is linked closely with an increased magnetic hyperthermia activity. The heating efficiency of commercial magnetic colloids has been reported to be between 0.15 and 3.12 nH m^2^/kg Fe [12]. Therefore, the magnetic-plasmonic nanoparticles in this study are comparable until thicker gold shells are added (R2 and R3).

## 4. Discussion

In this work, the following five stages of magnetic-plasmonic nanoparticles were studied: Fe_3_O_4_ nanoparticles (O), Fe_3_O_4_–Au core–satellite nanostructures (Os), Fe_3_O_4_–Au with thin gold shell (R1), Fe_3_O_4_–Au with intermediate thickness gold shell (R2), and Fe_3_O_4_–Au with thick gold shell (R3). These nanoparticle stages were studied in three applications—magnetophoresis, MRI or NMR contrasting, and magnetic hyperthermia.

**Figure 5:**
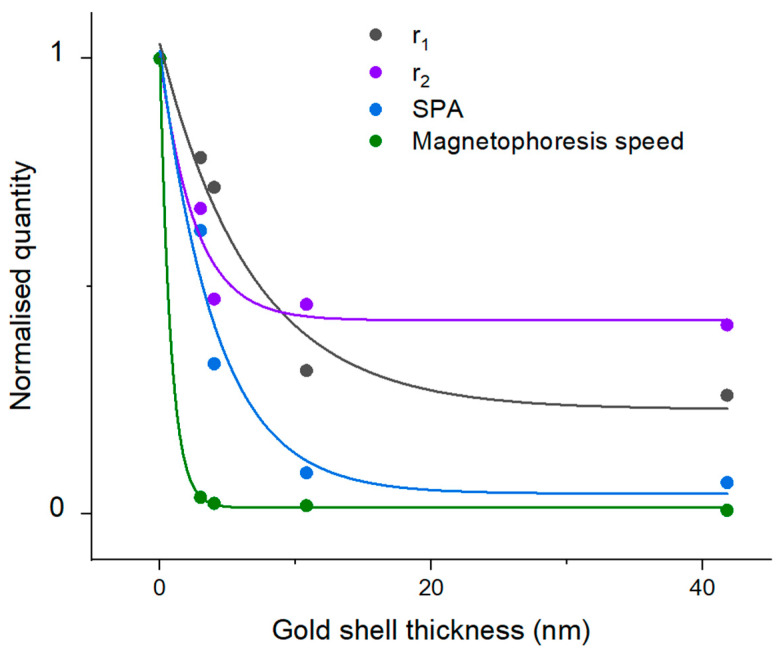
Exponential dependency of the normalized NMR relaxivities r_1_ and r_2_, the specific power absorption (SPA), and the magnetophoresis speed (v_mag_) as a function of gold shell thickness. See Table 1 for values.

The magnetophoresis rate of magnetic-based nanostructures is key in magnetic field-driven drug delivery, magnetic sorting, and other applications requiring magnetic manipulation. It was found that increased gold coating leads to a reduction in the magnetophoresis rate, rendering the nanoparticle less responsive to magnetic fields. The magnetophoresis speed is reduced to ca. 1% for the thickest gold shell compared to the uncoated iron oxide nanoparticle (see Table 1 for further details). The magnetophoresis speed decreases exponentially (see Figure 5) with increased gold shell thickness; thus, if responsivity is required for the application, a thinner gold coating is preferable. We attribute this reduced responsivity of the increased gold shell thickness to a reduced magnetic saturation, loss of cooperative behavior, and increased drag forces.

Iron oxide nanoparticles are commonly used as MRI contrast agents, specifically as T_2_ agents. In this work, the T_1_ and T_2_ relaxivities (r_1_ and r_2_) of the various magnetic-plasmonic nanostructures were measured. As with the magnetophoresis, the T_1_ and T_2_ relaxivities, r_1_ and r_2_, decreased exponentially with additional gold growth (see Table 1). Compared to the purely magnetic counterpart, the thickest gold shell has a reduced relaxivity, to 22.2% and 41.6% for r_1_ and r_2_, respectively. These relaxivities also decreased exponentially (see Figure 5) with increased gold shell thickness (see Figure 5). We attribute this decreased relaxivity of thicker gold shells to reduced magnetic saturation, loss of cooperative behavior, and reduced exchange interaction with water nuclei.

Lastly, increasing the gold content of the magnetic-plasmonic hybrid nanoparticles led to an exponential decrease (see Figure 5) in the specific power absorption of the nanoparticles undergoing magnetic hyperthermia. A reduction to 14.3% was seen for the intrinsic loss power parameter, a measurement of heating efficiency that considers magnetic field strength and frequency, for the thickest gold shell on the magnetic core compared to the purely magnetic nanoparticle. This reduced heating efficiency with gold addition is likely a result of reduced cooperative behavior along with reduced magnetic saturation.

## 5. Conclusions

In conclusion, increasing the gold shell thickness has a significant reduction in the magnetophoresis rate, T_1_ and T_2_ contrast, and magnetic hyperthermia activity of iron oxide nanoparticles. A number of factors are likely responsible for this reduced activity with increased gold shell thickness. We hypothesize that less cooperation due to reduced dipole–dipole magnetic interaction forces and a reduced magnetic saturation owing to the decreased magnetic volume and possible diamagnetic magnetic shielding by the gold shell are predominantly responsible.

In a previous work ([28]), we demonstrated that the thinner gold shells (R1 and R2) exhibit a large spectral drift between the optical absorption (highest photothermal activity) and the optical scattering (strongest optical imaging contrast). We demonstrate in this work that these thinner gold shells are also better for magnetic-based applications. Hence, this work further emphasizes that a thick gold shell is not necessary to achieve a highly functional nanotheranostic nanoplatform. In other words, thinner gold coating appears preferable in an extending series of both magnetic- and light-based applications.

## Figures and Tables

**Table 1:** Overview of the key findings in this work for the five stages of nanoparticle. Percentages in brackets indicate the percentage as compared to the uncoated iron oxide nanoparticle.

Application	Fe_3_O_4_ (O)	Fe_3_O_4_ + Au Seeds (Os)	Fe_3_O_4_ + Thin Au Shell (R1)	Fe_3_O_4_ + Medium Au Shell (R2)	Fe_3_O_4_ + Thick Au Shell (R3)
Nanoparticle total diameter (nm) [28]	20.5 ± 1.3	O + ~3 nm seeds	28.5 ± 2.2	42.1 ± 10.3	103.7 ± 16.9
Shell thickness (nm) [28]	-	-	≈4	≈10.8	≈41.6
Zeta Potential (mV) [28]	0.0 ± 4.1	−10.0 ± 6.8	−27.3± 2.8	−22.4 ± 2.1	−22.1 ± 3.2
Magnetophoresis time (min)	12	326	498	636	1421
Magnetophoresis speed, v_mag_ (μm/s)	9.7	0.4 (4.1%)	0.2 (2.1%)	0.2 (2.1%)	0.1(1%)
T_1_ relaxivity r_1_ (mM/s)	0.9	0.7 (77.8%)	0.7 (77.8%)	0.3 (33.3%)	0.2 (22.2%)
T_2_ relaxivity r_2_ (mM/s)	32.7	21.9 (67%)	15.4 (47.1%)	15.1 (46.2%)	13.6 (41.6%)
Relaxivity ratio r_2_/r_1_	35.6	30.6 (86%)	23.3 (65.4%)	51.5 (144.7%)	55.5 (155.9%)
Max. temperature	3.4	2.1 (61.8%)	1.5 (44.1%)	0.3 (8.8%)	0.1 (2.9%)
Initial 60 s ΔT/Δt (m °C/s)	18.8	11.7 (62.2%)	6.2 (33%)	1.7 (9%)	1.3 (6.9%)
Specific power absorption (SPA) (W/g)	93.7	58.3 (62.2%)	30.9 (33%)	8.5 (9.1%)	6.5 (6.9%)
Intrinsic loss power parameter (ILP) (H m^2^/kg)	0.7	0.5 (71.4%)	0.2 (28.6%)	0.1 (14.3%)	0.1 (14.3%)

**Table 2:** Relaxation properties of select examples from the literature, including SiO_2_ shells of varied thicknesses and magnetically assembled structures. Relaxation properties of the magnetic-plasmonic nanoparticles studied in this work are shown in the last 5 lines.

Sample	r_1_ (mM^−1^ s^−1^)	r_2_ (mM^−1^ s^−1^)	r_2_/r_1_	Frequency (MHz)	Field (T)	Ref.
Fe_3_O_4_ (20 nm) @Au (5 nm)	-	181.35		- ^1^	3	[56]
Fe_3_O_4_ (20 nm) @Au (5 nm) + PEG ^2^		162.3		-	3	[56]
Fe_3_O_4_ 9 nm + 1 nm SiO_2_ shell		94		-	3	[70]
Fe_3_O_4_ 9 nm + 5 nm SiO_2_ shell		68		-	3	[70]
Fe_3_O_4_ 9 nm + 10 nm SiO_2_ shell		47		-	3	[70]
Fe_3_O_4_ 9 nm + 13 nm SiO_2_ shell		32		-	3	[70]
Fe_2_O_3_ ≈ 10 nm	32	≈228	≈7.1	r_1_ 20r_2_ ≈500	r_1_ 0.4r_2_ 11.7	[69]
Fe_2_O_3_ ≈10 nm + ≈2 nm SiO_2_ shell	11.2	≈100	≈8.9	“ ^3^	“	[69]
Fe_2_O_3_ ≈10 nm + ≈8 nm SiO_2_ shell	<2	≈64	≈32.0	“	“	[69]
Fe_2_O_3_ ≈10 nm + ≈15 nm SiO_2_ shell	<2	≈47	≈23.5	“	“	[69]
Fe_2_O_3_ ≈10 nm + ≈20 nm SiO_2_ shell	<2	≈38	≈19.0	“	“	[69]
Fe_2_O_3_ ≈10 nm + ≈28 nm SiO_2_ shell	<2	≈23	≈11.5	“	“	[69]
Fe_2_O_3_ ≈10 nm + ≈52 nm SiO_2_ shell	<2	≈15	≈7.5	“	“	[69]
Fe_2_O_3_ ≈10 nm + ≈67 nm SiO_2_ shell	<2	≈13	≈6.5	“	“	[69]
Cluster core–shell Fe_3_O_4_ 6 nm–APTES (≈96.6 nm total)	0.006	40.6	6766.7	300	7	[74]
Cluster core–shell Fe_3_O_4_ 6 nm–GPTMS ^4^ (≈22.0 nm total)	0.026	14.4	553.8	300	7	[74]
Cluster core–shell Fe_3_O_4_ 6 nm–TEOS ^5^ (≈66.6 nm total)	0.016	13.8	862.5	300	7	[74]
Fe_3_O_4_ (30 nm) with asymmetric surface chemistry (amine and thiol)	-	44.87		-	1.4	[73]
Nanochains of Fe_3_O_4_ (30 nm) with amine and thiol surface	-	101.05		-	1.4	[73]
Fe_3_O_4_ (11 nm)–CTAB ^6^	31.25 (13.69)	81.37 (82.18)	2.6 (6.0)	20 (60)	0.47 (1.41)	[71]
Fe_3_O_4_ (12 nm) @mSiO_2_ shell (50 nm total)	3.65 (1.31)	84.26 (92.13)	23.1 (70.3)	“	“	[71]
Fe_3_O_4_ (12 nm) @mSiO_2_ shell (75 nm total)	2.13 (0.97)	79.93 (87.54)	37.5 (90.3)	“	“	[71]
Fe_3_O_4_ (12 nm) @mSiO_2_ shell (95 nm total)	0.61 (0.31)	50.13 (55.44)	82.2 (178.8)	“	“	[71]
Fe_3_O_4_ (≈7.48 nm)	≈37	≈48	≈1.3	≈10	-	[75]
Fe_3_O_4_ (≈7.48 nm)–Au (5–8 nm) dimer	≈5	≈62	≈12.4	“	-	[75]
Au core (5–8 nm)–Fe_3_O_4_ shell (≈15.92 nm total)	≈27	≈41	≈1.5	“	-	[75]
Fe_3_O_4_ cluster (200 nm)	-	230.7		-	-	[55]
Fe_3_O_4_ cluster (200 nm) + 5 nm Au seeds	-	147.7		-	-	[55]
Fe_3_O_4_ cluster (200 nm) + 20 nm Au seeds	-	163.1		-	-	[55]
Fe_3_O_4_ cluster (200 nm) + 25 nm Au shell	-	158.2		-	-	[55]
Fe_3_O_4_	-	167		-	-	[76]
Fe_3_O_4_–Au (core–shell)	-	61.9		-	-	[76]
Fe_3_O_4_–Au (yolk–shell)	-	149.4		-	-	[76]
γ-Fe_2_O_3_ core–Au Shell ≈28.38 nm	≈8.82 (≈10.35)	≈4.04 (≈3.99)		- (532 nm light)	100 μT	[30]
20.5 ± 1.3 nm Fe_3_O_4_ (O)	0.92	32.70	35.55	60	1.5	This work
O + ≈3 nm Au seeds (Os)	0.72	21.94	30.56	“	“	“
O + ≈4 nm thick Au shell (R1)	0.66	15.43	23.27	“	“	“
O + ≈10.8 nm thick Au shell (R2)	0.29	15.06	51.48	“	“	“
O + ≈41.6 nm thick Au shell (R3)	0.24	13.58	55.48	“	“	“

^1^ Unknown quantity. ^2^ Polyethylene glycol. ^3^ Ditto marks indicate repeated values. ^4^ (3-Glycidyloxypropyl)trimethoxysilane. ^5^ Tetraethyl orthosilicate. ^6^ Cetyltrimethylammonium bromide.
